# Autophagy and hepatic lipid metabolism: mechanistic insight and therapeutic potential for MASLD

**DOI:** 10.1038/s44324-024-00022-5

**Published:** 2024-08-02

**Authors:** Sana Raza, Sangam Rajak, Paul M. Yen, Rohit A. Sinha

**Affiliations:** 1 Department of Endocrinology, Sanjay Gandhi Postgraduate Institute of Medical Sciences, Lucknow, 226014 India; 2 Laboratory of Hormonal Regulation, Cardiovascular and Metabolic Disorders Program, Duke-NUS Medical School, Singapore, 169857 Singapore

**Keywords:** Biochemistry, Cell biology, Endocrinology, Gastroenterology

## Abstract

Metabolic dysfunction-associated steatotic liver disease (MASLD) originates from a homeostatic imbalance in hepatic lipid metabolism. Increased fat deposition in the liver of people suffering from MASLD predisposes them to develop further metabolic derangements, including diabetes mellitus, metabolic dysfunction-associated steatohepatitis (MASH), and other end-stage liver diseases. Unfortunately, only limited pharmacological therapies exist for MASLD to date. Autophagy, a cellular catabolic process, has emerged as a primary mechanism of lipid metabolism in mammalian hepatocytes. Furthermore, preclinical studies with autophagy modulators have shown promising results in resolving MASLD and mitigating its progress into deleterious liver pathologies. In this review, we discuss our current understanding of autophagy-mediated hepatic lipid metabolism, its therapeutic modulation for MASLD treatment, and current limitations and scope for clinical translation.

## Introduction

Metabolic dysfunction-associated steatotic liver disease (MASLD) previously referred as non-alcoholic fatty liver disease (NAFLD) represents a hepatic manifestation of metabolic syndrome. It is commonly associated with obesity and poses a significant risk factor for diabetes mellitus, chronic kidney disease, and cardiovascular diseases^1,2^. Over the past decade, the global prevalence of MASLD has markedly increased^3^, yet to date, limited pharmacological treatment exists^4^ for this disease. The central basis of MASLD pathogenesis is the dysregulation of hepatic lipid metabolism, affecting lipid synthesis, storage, mobilization, partitioning, and catabolism within the liver^4–6^. Hepatic steatosis, an early hallmark of MASLD development, arises from a compensatory response of the liver to partition the increased pool of free fatty acids (FFAs) derived from the elevated hepatic influx from circulation or de novo lipogenesis into triacylglycerol (TAG)^5^ within cytosolic lipid droplets (LDs). However, prolonged intrahepatic TAG storage can lead to hepatic insulin resistance and injury via lipid peroxidation and may lead to the onset of metabolic dysfunction-associated steatohepatitis (MASH) associated inflammation and fibrosis^7^. Therefore, enhancing hepatic TAG lipolysis and its coupling to mitochondrial fatty acid oxidation (FAO) seems as an effective strategy to reduce hepatic steatosis in both humans and preclinical animal models^8–10^. Interestingly, a cellular degradation process, macroautophagy hereafter referred to as autophagy, has emerged as a pivotal mechanism for stimulating lipid catabolism and resolving hepatic steatosis in mammals^11^. Autophagy-mediated lipid turnover, termed “lipophagy,” involves the engulfment of TAGs within autophagosomes, their delivery to lysosomes, and subsequent degradation by lysosomal acid lipase (LAL)^12^. Furthermore, lifestyle modifications and pharmacological stimulation of lipophagy show promising results in mitigating MASLD progression in preclinical animal models^11^. In addition to lipophagy, other types of autophagy and autophagy-related (ATG) proteins can also influence multiple aspects of hepatic lipid metabolism such as lipid droplet assembly, lipotoxicity, and mitochondrial homeostasis^13^. This review comprehensively examines the intricate interplay between autophagy and hepatic lipid metabolism, provides mechanistic insights into autophagy dysfunction in MASLD, and explores therapeutic avenues to combat MASLD via autophagy modulation. Understanding these molecular mechanisms holds promise for the development of targeted interventions to alleviate the burden of MASLD on global health.

## Molecular regulation of autophagy

**Fig. 1:**
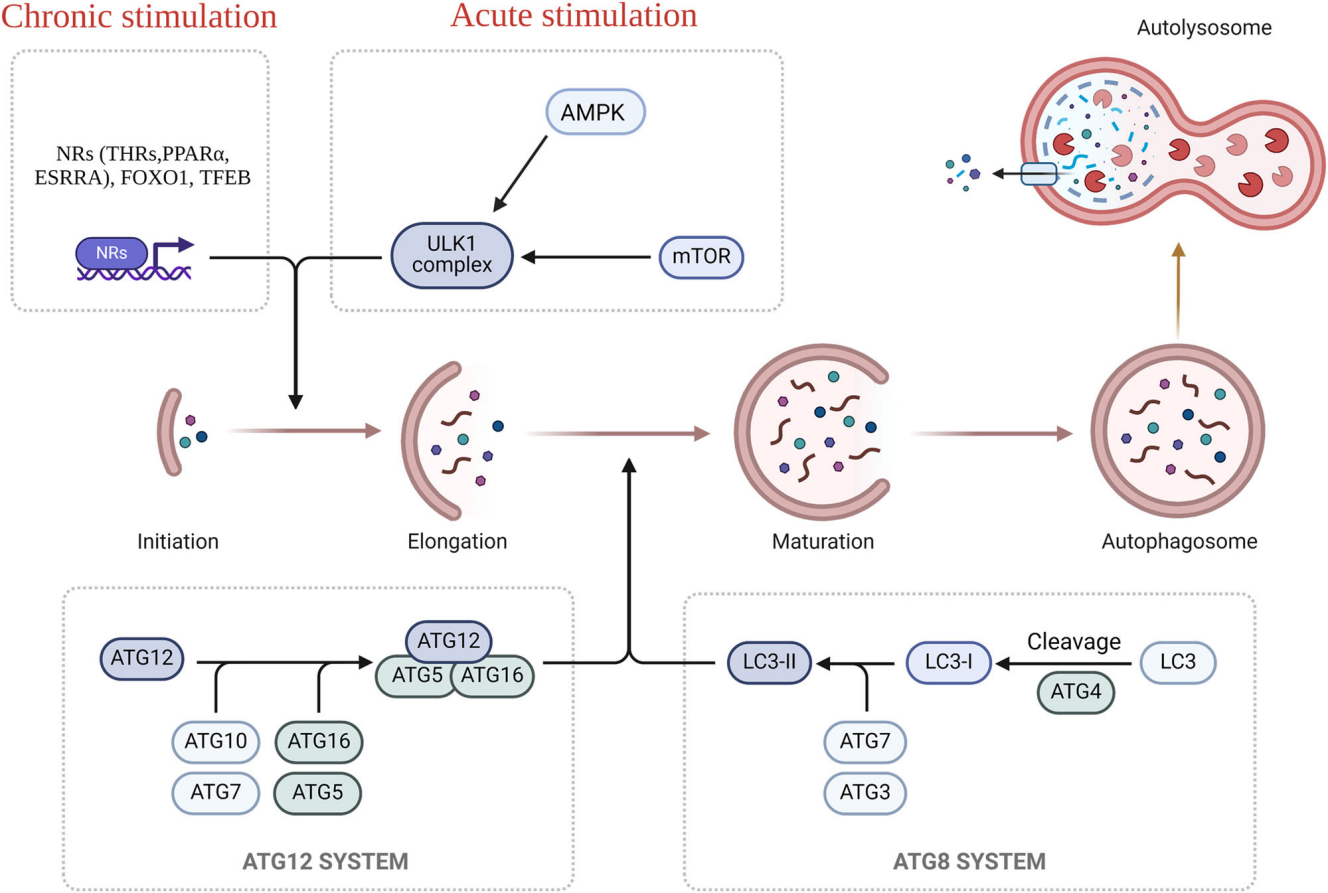
Molecular regulation of autophagy. Mammalian autophagy can be induced by both rapid kinase action (acute stimulation) or by transcriptional mechanism which require action of nuclear receptors (chronic stimulation). Upon induction, autophagic process involves a series of steps which includes several stages, such as initiation, which is the nucleation step resulting in the formation of a double-membraned structure known as the phagophore and this mechanism is facilitated by ULK1 complex and other proteins including Beclin 1. Following initiation, the phagophore increases in size by engulfing damaged cytosolic components to form a fully developed autophagosome. This step involves the conjugation of ATG5 with ATG12, facilitated by ATG7 and ATG10, to form the ATG12-ATG5 conjugate. Additionally, the microtubule-associated protein 1 light chain 3 (LC3) is cleaved by ATG4 to form LC3-I, which is further conjugated with phosphatidylethanolamine (PE) to form LC3-II, facilitating the maturation and packing of the autophagosome Further, the autophagosomes fuse with lysosomes, forming an autolysosome. This fusion allows the cargo to undergo degradation by lysosomal enzymes, releasing amino acids, fatty acids, and glucose that can be reused by the cell. After degradation, the resulting macromolecules are transported out of the autolysosome into the cytoplasm for recycling and reuse in cellular processes. This figure was created with BioRender.com.

Autophagy is a fundamental cellular process that entails the delivery of intracellular components for lysosomal degradation^14^. There are several types of autophagy that involve different modes of delivery to the lysosomes. Macroautophagy, involves the delivery of cellular components to lysosomes via a double membrane structure known as autophagosomes. Other forms of autophagy include Chaperone-mediated autophagy (CMA) which involves heat-shock proteins to deliver a cargo to lysosomes, Microautophagy mediates cargo delivery by invaginations or protrusions of the lysosomal membrane^14^, and lastly, Crinophagy deliver cellular cargo by secretory vesicles to lysosomes^15^. Among these, macroautophagy, is the most commonly occurring and most extensively studied in hepatic metabolism and is commonly referred to as “autophagy”. In the liver, autophagy serves two critical functions. Firstly, it provides amino acids during starvation by functioning as a non-selective bulk degradative process under nutrient-deprived conditions^16^. Secondly, autophagy maintains cellular homeostasis by selectively removing damaged organelles even during nutrient-sufficient states; thus, contributing to cellular health and rejuvenation^17^. Both selective and non-selective autophagy share common molecular machinery for autophagosome formation and fusion with lysosomes^18^, including the ATG family of proteins responsible for various stages of autophagy, such as induction, autophagosome assembly and elongation, docking, and fusion with lysosomal membranes, and autophagolyosome-mediated breakdown and release of autophagosomal contents^18^ (Fig. 1). The regulation of autophagy involves both acute (non-transcriptional) and chronic (transcriptional) mechanisms (Fig. 1). Acute regulation is mediated by nutrient- and energy-sensing kinases such as the mechanistic target of rapamycin complex 1 (MTORC1) and AMP-activated protein kinase (AMPK)^19^ (Fig. 1). Mechanistically, both MTORC1 and AMPK differentially regulate the activity of the key autophagic protein, Unc-51-like kinase 1 (ULK1) through its phosphorylation. MTORC1 can acutely inhibit while AMPK can activate ULK1 activity in cells, thereby regulating autophagy^20^. When nutrients are abundant, MTORC1 is active, suppressing autophagy. AMPK, on the other hand, is activated under conditions of low energy, such as during nutrient deprivation or exercise. AMPK, activates autophagy by phosphorylating ULK1 directly, promoting its activity. Chronic regulation involves the transcription of autophagosome- and lysosome-related genes by nuclear transcription factors in order to sustain the autophagic machinery and flux during prolonged stress conditions^21^ such as starvation (Fig. 1). Key transcriptional inducers of autophagy and lysosomal biogenesis include forkhead box protein O1(FOXO1)^22^, transcription factor-EB (TFEB)^23^ and nuclear hormone receptors such as peroxisome proliferator-activated receptors (PPARs)^24^, estrogen-related receptor alpha (ESRRA)^25^ and thyroid hormone receptors (THRs)^26^. These transcription factors coordinate the expression of genes encoding components of the autophagic machinery, ensuring its sustained activity and functionality during prolonged stress conditions.

## Autophagy and its role in TAG lipolysis

**Fig. 2:**
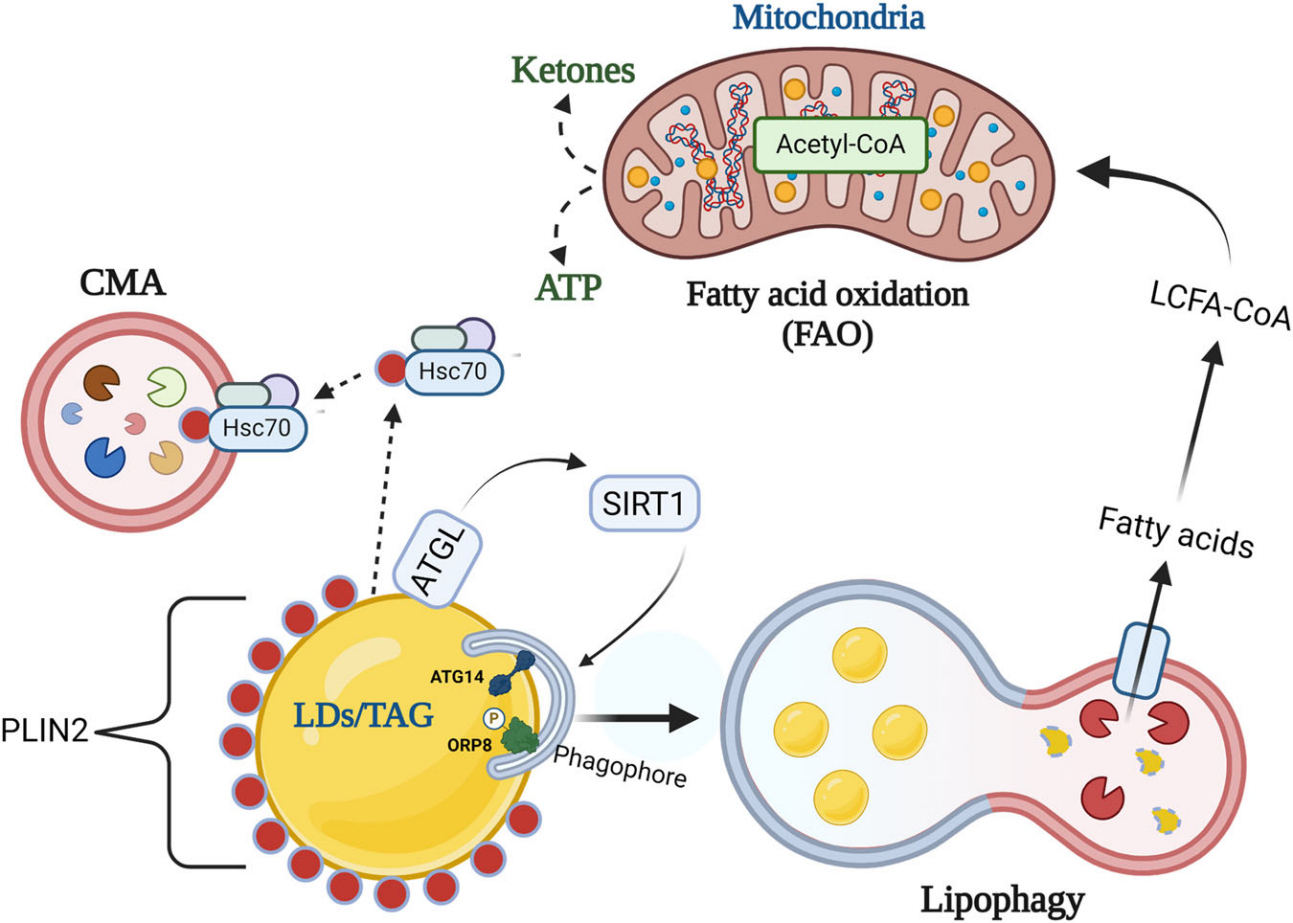
Lipophagy mediates intrahepatic TAG lipolysis. Lipophagy is a major mechanism of TAG lipolysis in hepatocytes wherein, with the facilitation of CMA and cytosolic lipase, ATGL, autophagosome engulf cytosolic TAG-containing LDs and fuse with lysosomes to release fatty acids by the action of LAL resident with the acidic milieu of lysosomes. The fatty acids released therein enter mitochondrial fatty acid oxidation (FAO) releasing ATP and ketones. This figure was created with BioRender.com.

Early evidence that linked autophagy to intrahepatic lipid catabolism came from studies implicating autophagy in the degradation of LDs associated with ApoB degradation^27^. However, a more definitive role of autophagy in TAG degradation was later demonstrated by Singh et al.^28^ using genetic and pharmacological approaches to show that impairment of autophagy during starvation leads to increased steatosis in hepatocytes. They further showed that TAG-containing LDs are directly engulfed by autophagosomes and subsequently degraded in lysosomes via lysosomal acid lipase (LAL) (Fig. 2). This process, termed “lipophagy”, is regulated by several intracellular kinases such as MTORC1 and AMPK. In hepatocytes, phosphorylation of perilipin 3 (PLIN3)^29^ (MTORC1 target) and ORP8^30^ (AMPK target) results in their recruitment to LDs, stimulating hepatic autophagy. Lipophagy is also facilitated by GTPases^31–33^, proteins involved in endo-lysosomal function^34^, N-degron pathway^35^, and calcium binding proteins^36^. Lipophagy is regulated by various nuclear transcriptional factors that respond to hormone and nutrient changes in the cellular environment. THRs were the first nuclear hormone receptors that were shown to induce hepatocyte lipophagy^37^. Thyroid hormone (TH) binds to THRs which then bind to the promoters of genes involved in lipid catabolism and stimulate their transcription. This transcriptional effect by TH is lost in mice expressing mutant THRs that are unable to bind TH^37^. TH induction of lipophagy is essential for increasing mitochondrial fatty acid oxidation (FAO) and ketogenesis, and genetic silencing of the autophagic gene, *ATG5*, blunts TH-induced ketogenesis in mice^37^. Additionally, TH stimulates the expression of chromosome 19 open reading frame 80 (C19orf80), located on lipid droplets or within the lysosome-associated compartment in cells, and is essential for TH-induced lipophagy in hepatic cells^38^. The nutrient-sensing transcription factors, PPARα and FXR, also regulate hepatic autophagy^24^. Notably, both PPARα in a fasted state and FXR in a fed state compete for binding on the autophagy gene promoters, with PPARα stimulating hepatic lipophagy in a fasted state and FXR inhibiting it in the fed state^24^. Another important transcription factor regulating hepatic lipophagy is TFEB, which translocates to the nucleus in response to MTORC1 inhibition and binds to the promoter of several autophagy and lysosomal genes to enhance their transcription^39^. The liver-X receptor α (LXRα) indirectly regulates hepatic lipophagy by modulating the expression of gene regulatory RNAs, modulating the expression of lipophagy genes^40^.

The interplay between CMA and macroautophagy/lipophagy during TAG catabolism has been well established^41^. LD‐resident perilipin proteins PLIN2 and PLIN3 act as barriers against lipophagy-mediated LD engulfment, and directly interact with the CMA-specific chaperone protein, heat shock cognate 70 (Hsc70), before undergoing lysosomal degradation^41^. Interestingly, mutation of the pentapeptide motif in PLIN2 recognized by Hsc70 leads to LD accumulation, suggesting the removal of the perilipin coat surrounding LDs is a prerequisite for lipophagy^41^ (Fig. 2). PLIN3 helps to activate lipophagy by functioning as a docking protein that regulates the assembly of autophagic complexes on LD, which is dependent on mTORC1.

Similarly, crosstalk exists between cytosolic lipase, adipose triglyceride lipase (ATGL), and lipophagy^42^. Overexpression of ATGL increases hepatic lipophagy and involves the activation of Sirtuin‐1 (SIRT1), a nicotinamide adenine dinucleotide‐dependent protein deacetylase^43^. Although lipophagy primarily supplies intracellular free fatty acids (FFAs) for FAO, recent findings indicate that it also may facilitate extracellular lipid secretion by hepatocytes to mitigate fatty acid-induced lipotoxicity^44^ (Fig. 2).

Recently, identification of putative lipophagy receptors has demonstrated lipophagy to be a highly selective autophagy process in mammalian cells. In this context, a lipid transfer protein ORP8 located on LDs has been shown to act as a lipophagy receptor in hepatocytes^30^. Notably AMPK mediated activation of ORP8 enhances its direct interaction with phagophore-anchored LC3/GABARAPs thereby promoting lipophagy^30^ (Fig. 2). More recently, autophagy protein ATG14 has been shown to act as a selective lipophagy receptor to assist lipid droplet degradation^45^ (Fig. 2).

Although most of our understanding of lipophagy comes from the studies performed on hepatocytes, lipophagy has been documented to affect the cellular function of other hepatic cells namely hepatic stellate cells (HSCs). Lipophagy has been shown to promotes the decomposition of LDs and accelerates the activation of HSCs^46^. This eventually leads to the progression of liver fibrosis^46^.

Thus, the regulation of lipophagy is a complex yet promising area of research to counteract MASLD.

## Autophagy and its role in mitochondrial fatty acid oxidation

**Fig. 3:**
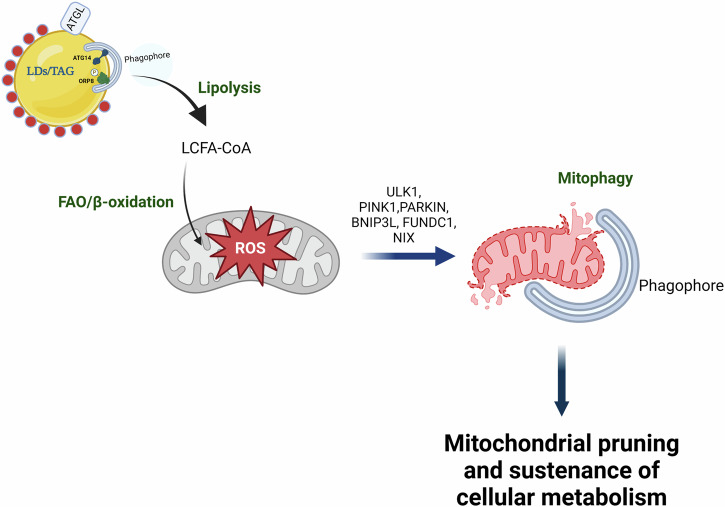
Mitophagy executes mitochondrial pruning to sustain hepatic FAO. Mitophagy is a mechanism wherein the damaged mitochondrial pool resulting from a high rate of oxidative stress from FAO are eliminated via lysosomes. This quality control mechanism ensures that a healthy pool of mitochondria can be maintained for sustaining fuel utilization and ATP production without compromising cellular health. Mitochondrial priming is mediated by several mitochondrial proteins (PARKIN/PINK1, BNIP3, NIX, and FUNDC1) which give an “eat me signal” to autophagosomes and helps in their recognition and engulfment within autophagososmes. This figure was created with the Mind the Graph platform.

FFAs, primarily long-chain fatty acids (LCFA) which are released by cytosolic or lysosomal lipase activity are activated via conjugation with Coenzyme A (CoA), a reaction catalyzed by Acyl-CoA synthetases (ACS) and shuttled by CPT1α to the mitochondria for fatty acid oxidation (FAO)/β-oxidation. This process reduces the intracellular accumulation of TAGs in hepatocytes as well as generates NADH and FADH_2_ required for mitochondrial oxidative phosphorylation. However, stimulation of FAO is associated with the generation of reactive oxygen species (ROS) that cause mitochondrial damage and apoptosis^47^. Autophagy serves to identify and remove damaged mitochondria through a process known as “mitophagy”^48^. Similar to lipophagy, mitophagy employs the core macroautophagy machinery in the steps just prior to autophagosome-lysosome fusion (Fig. 3). However, early mitophagy events such as priming of damaged mitochondria in hepatocytes require translocation/activity of specific mitophagy proteins, including Unc-51-like kinase (ULK1), Parkin, BCL-2/adenovirus EIB 19-kDa interacting protein (BNIP3), NIX and FUNDC1^48^ (Fig. 3). Of note, ULK1 translocation to damaged mitochondria regulates TH-induced mitophagy^49^. Interestingly, loss of ULK1 not only impairs TH-induced mitophagy but also reduces mitochondrial activity in response to TH stimulation in human hepatic cells^50,51^. In further support of the critical role of mitophagy in FAO, PINK1/Parkin are implicated in reducing hepatic steatosis in a MASLD animal model^49,52^. BNIP3 also exerts a protective effect on MASLD pathogenesis, and loss of BNIP3 results in impaired FAO and increased steatosis in animal models of MASLD^53^. Furthermore, the nuclear orphan receptor, RORα, increases BNIP3 expression to maintain mitochondrial quality during nutrient-replete conditions in an animal model of MASLD^54^. Taken together, these studies underscore the importance of mitophagy in maintaining mitochondrial health and sustaining FAO in hepatic cells (Fig. 3).

## Autophagy and its role in hepatic TAG assembly

Although the role of lipophagy in TAG lipolysis is well established, several studies suggest an intriguing paradoxical role of autophagy/autophagy-related proteins in promoting TAG assembly. Shibata et al., initially demonstrated that autophagy protein microtubule-associated protein 1A/1B light chain 3B (MAP1LC3B) plays a role in LD formation during starvation, and this LD assembly is impaired in autophagy-deficient hepatocytes^55^. In this connection, lipidated MAP1LC3B (LC3B-II) colocalizes with LDs during starvation, and genetic silencing of MAP1LC3B hinders LD formation and expansion in hepatocytes^56^. Similarly, loss of another autophagy protein, FIP200, impairs the ability of hepatocytes to store TAG/LDs when the animals are challenged with hepatosteatotic diet^57^. More recently, S100 calcium binding protein A11 (S100A11), was found to be upregulated in the livers in animal models of MASLD and patients, where it increased hepatic lipogenesis and TAG accumulation by inducing autophagy^58^. This pro-lipogenic effect of S100A11 is lost when autophagy is inhibited in hepatocytes^58^. These findings suggest that there may be pleiotropic roles for hepatic autophagy with respect to TAG catabolism and anabolism. The latter may be due to the non-autophagic effects of certain autophagy proteins or context-specific relationships between autophagy and lipid metabolism within the hepatocytes. Unexpectedly, genetic inhibition of autophagy in extrahepatic tissues such as skeletal muscle and white adipose tissue WAT can protect hepatocytes from steatosis in mice fed a high-fat diet via inter-organ crosstalk^59,60^. These intriguing findings are related to the increased production of FGF21 from autophagy-deficient muscles, which alleviates hepatic insulin resistance and enhances lipid metabolism in mouse-fed high-fat diet (HFD)^59^. Similarly, the ablation of autophagy genes in WAT suppresses the levels of circulating FFAs thereby preventing hepatic steatosis in mice fed HFD^60^. When these findings are taken together, further studies are warranted to fully elucidate these apparently paradoxical effects of autophagy and autophagy proteins on hepatic TAG content.

## Autophagy and transcription regulation of hepatic lipid metabolism

**Fig. 4:**
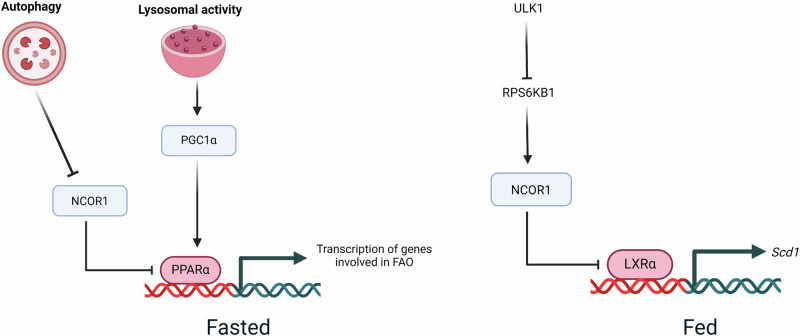
Autophagy and autophagy-related family proteins regulate transcriptional activity on the promoter of genes involved in hepatic lipid metabolism. Regulation of cellular transcription by autophagy or autophagy-related proteins is another mechanism of hepatic lipid metabolism. Under a fasted state, autophagy degrades the nuclear corepressor NCOR1 thereby preventing its inhibition of PPARα driven transcription of genes involved in mitochondrial FAO. Additionally, autophagy-lysosomal mediated increase in PGC1α levels further potentiate PPARα transcriptional activity under a fasted state. However, under nutrient-sufficient conditions autophagy-related protein ULK1 increases hepatic lipid anabolism by promoting LXRα driven transcription of lipogenic gene SCD1. This effect of ULK1 is autophagy-independent and is mediated by its inhibitory phosphorylation of RPS6KB1 which regulates NCOR1 nuclear localization. This figure was created with BioRender.com.

Apart from directly targeting cellular organelles like LDs and mitochondria to regulate hepatic lipid metabolism, autophagy can also affect the transcription of lipid metabolic genes. In a study performed in fasted conditions, autophagy was shown to degrade a nuclear corepressor, NCOR1 which negatively regulates the expression of PPARα target genes^61^ (Fig. 4). Since, PPARα binds to the promoter of several FAO genes and including its own gene promoter, therefore loss of NCOR1 increased hepatic lipid catabolism under fasting condition. concurrently, loss of autophagy inhibited fasting-induced FAO via repressed PPARα signaling^61^ (Fig. 4). Additionally, lysosomal dysfunction and TFEB silencing also decreased the protein levels of PPARα coactivator PGC1α^62^ (Fig. 4). Therefore, autophagy may act as a fine tuner of PPARα transcriptional activity by modulating the relative levels of NCOR1 and PGC1α^63^ (Fig. 4).

Interestingly, under nutrient-sufficient conditions autophagy-independent action of autophagy-related protein ULK1 increases TAG assembly in hepatocytes as an adaptative response to saturated fatty acid influx (Fig. 4). This process involves the direct interaction of ULK1 and ribosomal protein S6 kinase B1 (RPS6KB1), to reduce the nuclear shuttling of nuclear corepressor, NCoR1 and increases liver-X-receptor (LXR)-mediated transcription of the pro-lipogenic gene, stearoyl-CoA desaturase 1 (SCD1)^45^ (Fig. 4). Furthermore, ULK1 positively regulates cholesterol biosynthesis pathway in hepatocytes through an autophagy-independent regulation of FOXO3a transcriptional activity^46^.

Therefore, based on the nutrient abundance autophagy-related proteins may exert opposing effect on hepatic lipid metabolism and may involve autophagy dependent or independent regulation on nuclear transcriptional networks.

## Autophagy dysregulation in MASLD

Given the profound effects of autophagy on hepatic lipid metabolism, it is perhaps not surprising that it is dysregulated in MASLD. Indeed, initial studies performed in dietary and genetic animal models of MASLD demonstrate dysregulated hepatic autophagy. It appears that acute exposure to a high-fat diet may induce hepatic autophagy as an adaptative response to limit lipotoxicity^64,65^; however, chronic hepatic fat accumulation inhibits autophagy in hepatocytes^13,66–69^, The mechanism(s) underlying the differential dynamics of intrahepatic autophagy regulation remains unclear but may be related to the relative expression and activity of autophagy regulators such as MTORC1 and AMPK in response to acute *vs*. chronic lipid exposure^64^. Suppression of autophagy after a prolonged high-fat diet may also result from differential changes in the sensitivities to exogenous hormones such as insulin^70^ and TH^50^. Autophagy inhibition in vitro and in vivo may involve lipid-induced perturbations occurring at several steps of the autophagy process. Firstly, both genetic and dietary models of obesity induce MASLD by markedly decreasing the expression of hepatic autophagy proteins such as MAP1LC3B, Beclin 1, Atg5, and Atg7 to cause an early block in autophagy^71^. Additionally, calpain 2-degrades Atg3 and Atg7 in the livers of mice with MASLD, and together with MTORC1 activation from inflammation^72^, has been proposed as another possible mechanism for the observed early block of autophagy^73^. Besides affecting the initiation and elongation process of autophagy, chronic lipid-loading also reduces autophagosome-lysosome fusion by altering the expression of proteins such as Rubicon^74^ and Syntaxin 17^75^, to cause a late block in autophagic flux. Late block in autophagic flux within hepatocytes also is induced by saturated fat-mediated permeabilization of lysosomal membrane and derangement of lysosomal function^76,77^.

Consistent with the data obtained from preclinical models of MASLD, autophagy dysregulation has also been observed in human MASLD^78,79^. Notably, elevated MAP1LC3 and p62/SQSTM1 levels along with ER-stress markers observed in severe MASLD liver biopsy indicate autophagic flux defects which possible results in increased cellular stress^80^. Additionally, recent clinical findings suggest that the severity of MASLD is associated with compromised hepatic FAO and reduced markers of mitophagy in the liver^81^. Taken together, these studies highlight the potential for autophagy to be a therapeutic target for MASLD.

## Autophagy modulators as treatment strategies for MASLD

MASLD represents a global health concern with limited therapeutic options. Given the potential role of autophagy in hepatic lipid metabolism, targeting autophagy represents a promising therapeutic approach for MASLD. Autophagy modulators represent a diverse class of compounds that can either inhibit or enhance the autophagic process. Several natural and synthetic molecules are known to modulate autophagy, including both nutritional and pharmacological supplements.

- **Pharmacological modulators of hepatic autophagy**

These include an array of compounds that modulate the autophagic process by targeting key signaling pathways and molecular targets involved in autophagy regulation. Here are some examples of pharmacological regulators of autophagy-

i. *Rapamycin, Carbamazepine*, *and Verapamil*: The first preclinical study to show that autophagy enhancers reduce hepatic steatosis in murine models of MASLD, used two autophagy inducers, carbamazepine and rapamycin^82^. Both carbamazepine and rapamycin improved hepatic steatosis, liver injury, and reduced serum triglyceride levels and insulin resistance in mice fed high fat^82^. Conversely, chloroquine, which is known to block autophagy, worsened hepatic steatosis and injury in these mice^82^. Additionally, pharmacological inhibition of calcium channels using the FDA-approved drug verapamil successfully restores autophagic flux and reduces hepatic lipid droplet accumulation, insulin resistance, and steatohepatitis in both in vitro and in vivo models of MASLD^83^ suggesting that repurposing of FDA-approved drugs which can also induce autophagy/lipophagy can be used for correction of MASLD pathologies however clinical trials in this respect are still awaited.
ii. *Metformin*: Metformin is an insulin-sensitizing agent and first-line antidiabetic drug. It induces autophagy through activation of the AMP-activated protein kinase (AMPK) pathway. In line with this, other specific AMPK activators, including A-769662^84^ and MK-8722^85^ also act as autophagy inducers. However, in ob/ob mice, metformin administration has been shown to reduce hepatosteatosis by increasing SIRT1 expression and inducing autophagy via a protein kinase A (PRKA)-independent pathway^86^.
iii. *Statins*: Statins are a class of lipid-lowering drugs that reduce cholesterol synthesis by inhibiting the rate-limiting enzyme in cholesterol biosynthesis, 3-hydroxy-3-methylglutaryl-coenzyme A (HMG-CoA) reductase. Statins also inhibit MTORC1 and promote autophagy in hepatocytes, causing increased degradation of lipid droplets which may help reduce hepatic steatosis. Some statins, such as Simvastatin or Atorvastatin also have been reported to activate AMPK, a key regulator of cellular energy homeostasis and autophagy^87^. In mice fed HFD, lovastatin and ezetimibe (L/E) co-treatment increases SREBP-2-mediated autophagy and reverses hepatic TG accumulation. The lovastatin-mediated increase in SREBP-2 also activates patatin-like phospholipase domain-containing enzyme 8 (PNPLA8) gene and reduces hepatic steatosis by increasing autophagy^88^. Notably, a recent cross-sectional investigation was performed within the Rotterdam Study and the PERSONS cohort showed that, indeed, statin use was associated with a lower prevalence of MASH and fibrosis and might prevent MASLD in humans^89^.
iv. *PPAR agonists*: PPARα agonists such as fibrates are potent inducers of hepatic autophagy^90^. Fenofibrates are effective in preventing hepatic steatosis by inducing TFEB-mediated lipophagy in hepatocytes^91^. PPARγ activation by pioglitazone leads to the upregulation of genes involved in lipid metabolism, insulin sensitivity, and autophagy. Through PPAR-γ activation, pioglitazone enhances autophagic flux in hepatocytes, promoting the clearance of lipid droplets and improving hepatic steatosis. Pioglitazone attenuates hepatic steatosis in mice by increasing cytosolic lipolysis, fatty acid β-oxidation, and autophagy in a PPARα-and PPARγ-dependent manner^92^.
v. *Tat-beclin 1* (Tat-BECN1):Tat-beclin-1, is a synthetic peptide derived from a region of the autophagy protein, Beclin 1, and the HIV-1 Tat protein to enhance cellular uptake. It stimulates hepatic autophagy and has been incorporated into nanoparticles for more targeted *in vivo* delivery to the liver in MASLD disease models. Early promising studies show that Tat-Beclin nanoparticles (NP T-B) can reduce intracellular lipid accumulation in a cellular model of MASLD^93^.

b. **Nutritional supplements**

Several nutritional supplements are potential autophagy modulators used to treat hepatic steatosis.

i. *Spermidine*: Spermidine, a naturally occurring polyamine, has been reported to induce autophagy by inhibiting histone acetyl transferases (HATs). Spermidine reduced hepatic lipid accumulation, insulin resistance, hepatic inflammation, and fibrosis in mice fed Western diet/fructose by hypusination of translation factor EIF5A to reduce mitochondrial protein expression and by increasing thyroid hormone-responsive protein (THRSP)-mediated autophagy^9,94^.
ii. *SIRT1 activators*: Resveratrol is a polyphenolic compound found in red wine and fruits and induces autophagy by multiple mechanisms including through SIRT1 activation. Resveratrol administration in rats fed high-fat diet decreased hepatic steatosis which was coupled with increase in hepatic SIRT1 expression and autophagy induction^95^.
iii. *Trehalose*: Trehalose, a disaccharide sugar found in, bacteria, fungi, insects, and plants, is an autophagy inducer. Trehalose attenuates liver steatosis, in part, by inhibiting hepatic glucose uptake by SLC2A/GLUT (solute carrier family 2 /facilitated glucose transporter) to create a starvation-like state that stimulates autophagic flux^96,97^. Oral administration of trehalose induces autophagy and reduces hepatosteatosis in apolipoprotein E knockout (apoE−/−) mice^98^.
iv. *Caffeine*: Caffeine is a widely consumed stimulant found in coffee, tea, beverages, and supplements. Caffeine induces autophagy, increases lipid uptake in lysosomes, and markedly reduces hepatic steatosis in mice fed HFD^99^. In a meta-analysis based on 11 epidemiological studies, the risk for MASLD was significantly lower in subjects who consumed coffee than those who did not (pooled RR value of 0.77 (95% CI 0.60–0.98)^100^.
v. *Green tea*: Epigallocatechin gallate (EGCG), the active component of green tea, is a polyphenol known for its anti-inflammatory and antisteatotic hepatic effects. EGCG induces hepatic autophagy by promoting autophagosome formation and stimulating autophagic flux via increased phosphorylation of AMPK^101^. In humans double-blind placebo-controlled study showed beneficial effects of green tea catechins in resolving MASLD pathogenesis^102^.

c. **Hormones and vitamins**
  Hormones and vitamins play critical roles in regulating cellular metabolism, and their dysregulation has been linked to the development of metabolic disorders such as MASLD. In animal models of MASLD, exogenous administration of certain hormones, their metabolites, or hormone mimetics effectively reduces lipotoxicity. In particular, thyroid hormones (THs) and their metabolites have potent pro-autophagy effects in vivo, by reducing lipotoxicity and hepatic lipid accumulation in hepatocytes^26,37^. Recently, liver-directed and THRβ-selective agonist, Resmetirom, became the first drug to obtain US Food and Drug Administration approval for NASH treatment [Clinical Trial: NCT03900429]. Other hormones such as epinephrine^103^, glucagon-like peptide-1 (GLP-1)^104^, ghrelin^105^, and fibroblast growth factor 21 (FGF21)^106^ also induce autophagy in hepatic cells and exhibit antisteatotic properties in vivo. Besides hormones, vitamins also induce hepatic autophagy and reduce lipotoxicity in preclinical models of MASLD^107^. Both 25- and 1,25-dihydroxyvitamin D increase ATG16L1 expression and autophagy to attenuate hepatosteatosis in mice fed HFD^108^. Similarly, vitamin B12 and folate supplementation enhance autophagic flux in hepatocytes, and reduce hepatic inflammation and fibrosis in mice fed Western diet/fructose to induce MASH^75^.
d. **Lifestyle-related changes**

Several non-pharmacological interventions, such as diet, exercise, and intermittent fasting stimulate hepatic autophagy. Lifestyle interventions exert profound effects on hepatic autophagy and contribute to the management of MASLD by promoting lipid metabolism, mitochondrial quality control, and cellular homeostasis in the liver. Various studies have demonstrated the benefits of moderate and longtime exercise on inducing hepatic lipophagy and mitophagy to enhance hepatic lipid clearance in animal models of MASLD^109–114^. Similarly, intermittent fasting regimens such as time-restricted feeding, induce hepatic autophagy and TAG lipolysis in preclinical models of MASLD^115–117^. However, the relative contribution of autophagy in the observed benefits of lifestyle-related changes remains to be investigated.

## Discussion

Autophagy plays an important and central role in hepatic lipid metabolism and its dysfunction is a major contributor to the pathogenesis of MASLD. The increasing prevalence of MASLD around the globe and the lack of many pharmacological options make autophagy an attractive target for the treatment of MASLD and MASH. However, there are certain knowledge gaps that need to be filled before initiating clinical trials with autophagic modulators^118^. Firstly, there is a need to identify reliable non-invasive autophagic flux biomarkers in man to assess the efficacy of autophagic modulators in humans. This is very important to assess the efficacy of certain FDA-approved drugs as autophagy inducers in clinical trials. It is possible that specific metabolites such as ketones or other FAO byproducts can serve as readouts of hepatic lipophagy but more validation is required. Secondly, liver-specific delivery of autophagic inducers needs to be developed to avoid or minimize extra-hepatic side effects since the induction of autophagy in other tissues may have deleterious effects. Finally, and most importantly, the effect(s) of autophagy modulation in different liver cells^119^ (e.g., hepatocytes, stellate cells, Kupffer cells, and cholangiocytes) and the stage-specific dynamics of autophagy during MASLD progression need to be better understood. However, despite these limitations, the beneficial metabolic effects of hepatic autophagy are evident and show promise as they potentially can be harnessed for the treatment of MASLD.
